# Effect of Salt Stress on the Expression and Promoter Methylation of the Genes Encoding the Mitochondrial and Cytosolic Forms of Aconitase and Fumarase in Maize

**DOI:** 10.3390/ijms22116012

**Published:** 2021-06-02

**Authors:** Alexander T. Eprintsev, Dmitry N. Fedorin, Mikhail V. Cherkasskikh, Abir U. Igamberdiev

**Affiliations:** 1 Department of Biochemistry and Cell Physiology, Voronezh State University, 394018 Voronezh, Russia; bc366@bio.vsu.ru (A.T.E.); rybolov@mail.ru (D.N.F.); fess-ru@mail.ru (M.V.C.); 2 Department of Biology, Memorial University of Newfoundland, St. John’s, NL A1B 3X9, Canada

**Keywords:** aconitase, fumarase, NaCl, promoter methylation, salt stress, *Zea mays* L.

## Abstract

The influence of salt stress on gene expression, promoter methylation, and enzymatic activity of the mitochondrial and cytosolic forms of aconitase and fumarase has been investigated in maize (*Zea mays* L.) seedlings. The incubation of maize seedlings in 150-mM NaCl solution resulted in a several-fold increase of the mitochondrial activities of aconitase and fumarase that peaked at 6 h of NaCl treatment, while the cytosolic activity of aconitase and fumarase decreased. This corresponded to the decrease in promoter methylation of the genes *Aco1* and *Fum1* encoding the mitochondrial forms of these enzymes and the increase in promoter methylation of the genes *Aco2* and *Fum2* encoding the cytosolic forms. The pattern of expression of the genes encoding the mitochondrial forms of aconitase and fumarase corresponded to the profile of the increase of the stress marker gene *ZmCOI6.1.* It is concluded that the mitochondrial and cytosolic forms of aconitase and fumarase are regulated via the epigenetic mechanism of promoter methylation of their genes in the opposite ways in response to salt stress. The role of the mitochondrial isoforms of aconitase and fumarase in the elevation of respiration under salt stress is discussed.

## 1. Introduction

Aconitase (aconitate hydratase; citrate (isocitrate) hydro-lyase (*cis*-aconitate-forming), EC 4.2.1.3) and fumarase (fumarate hydratase, EC 4.2.1.2) are the tricarboxylic acid (TCA) cycle enzymes catalyzing the equilibrium correspondingly between citrate and isocitrate and fumarate and malate. They are localized in both mitochondria and cytosol [1,2,3,4] and participate in the interchange of the TCA cycle intermediates and their conversion in both compartments. The operation of the two forms of fumarase can support the interchange of organic acids via the malate valve [5], while the distribution of aconitase between the two compartments provides the conditions for operation of the citrate valve [6,7]. The latter contributes to the reduction of NADP^+^ in the cytosol, supplies 2-oxoglutarate for amino acid biosynthesis [7], and stimulates the expression of alternative oxidase [8]. 

The efflux of citrate from mitochondria is facilitated by the inhibition of isocitrate conversion at high redox level [9] and by the inhibition of aconitase by reactive oxygen species (ROS) and reactive nitrogen species (RNS) [10,11]. Schwarzländer et al. [12] demonstrated that abiotic factors such as salinity modulate the redox level in mitochondria and trigger the production of ROS. To date, most of the studies, however, are focused on the photosynthetic response to salinity, while less consideration has been provided to the effect of salt stress on plant respiration and on the involvement of mitochondria [13]. The phenomenon of “salt respiration” was described many decades ago [14]. It was explained by the adaptive function of respiratory metabolism in the adaptation to salinity; however, the concrete metabolic processes underlying these changes in respiratory rate require further clarification. The observed changes necessarily include regulation by the conditions of salinity of the enzymes and transporters involved in respiration at the transcriptional, translational, and post-translational levels. 

The sodium signaling wave results in the elevation of Ca^2+^, triggers ROS formation and stimulates ion transport between tissues, cells, and cellular compartments [15]. This directly affects the TCA cycle activity and influences tri- and dicarboxylic acid exchange between mitochondria and cytosol [16]. The processes associated with salt treatment lead to an increase in TCA cycle intermediates [17,18], while the mitochondrial respiration shifts from the TCA cycle to the GABA shunt [19], and the noncoupled pathways of electron transport are induced [20,21]. It is important to explore how this can be related to a differential expression of the mitochondrial and cytosolic forms of the TCA cycle enzymes. The changes in expression of corresponding genes may have important consequences for the intercompartmental interactions in the stressed plant cells and result in the observed shifts in metabolism. There are several indications that salt stress affects DNA methylation and causes other epigenetic changes, including modifications of histones [22,23], in particular, depending on the salinity tolerance [24], and the level of methylation under salt stress can be mediated by phytohormones such as brassinosteroids [25]. The identified genes affected by methylation under NaCl treatment include those that participate in cell cycle, development, and different metabolic processes [26]. However, there is no sufficient information about the epigenetic changes linked to respiratory metabolism in the conditions of salinity. 

In the current investigation, we studied the effect of NaCl on the expression and promoter methylation of the genes encoding the mitochondrial and cytosolic forms of aconitase and fumarase in maize seedlings. It has been shown that, while the expression of the mitochondrial forms of aconitase and fumarase is stimulated by the salt stress, the expression of the cytosolic forms of these enzymes is suppressed. This regulation takes place via the methylation of promoters of corresponding genes and results in the corresponding changes in activities of aconitase and fumarase in both compartments. These changes may result in the tighter connection of the TCA cycle with mitochondria in stress conditions, leading to the redistribution of organic acids between mitochondria and cytosol and to the shift in the malate and citrate valves operation upon the application of NaCl to plants. 

## 2. Results

### 2.1. Analysis of CpG Islands

**Figure 1:**
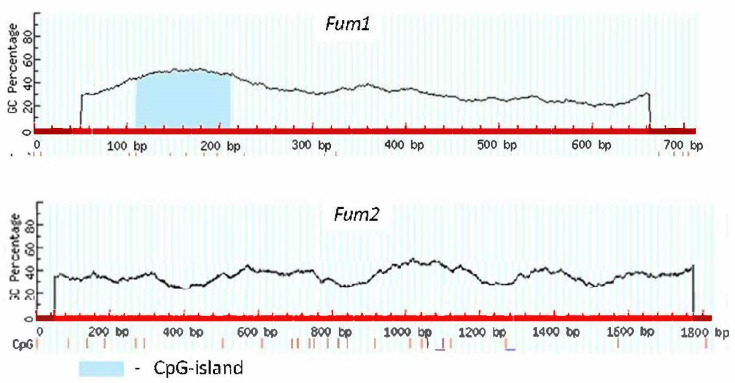
Analysis of CG dinucleotides in the promoters of the *Fum1* and *Fum2* genes from maize. The position of CG nucleotides is indicated by vertical lines.

For the results of the analysis of CpG islands in the promoters of the genes encoding aconitase using the software MethPrimer (LiLab, UCSF, San Francisco, CA, USA, https://www.urogene.org/methprimer/, accessed on 8 February 2018), we refer to our previous publication [27]. The results of the analysis of CpG islands in the promoters of the genes encoding fumarase are presented in Figure 1. As shown earlier [27], the gene *Aco1* is characterized by the presence in its promoter of the two CpG islands of the sizes of 105 and 361 bp, while the promoter of the gene *Aco2* contains one island of 170 bp. The genes *Fum1* and *Fum2* encoding, correspondingly, the mitochondrial and the cytosolic fumarase [3] show a different pattern of CpG islands. The promoter of the gene *Fum1* contains one island of 102 bp, while the gene *Fum2* is characterized by the absence of CpG islands in its promoter. This does not mean the absence of the epigenetic mechanism of its regulation but indicates the tissue-specific standard mechanism of methylation of CG dinucleotides, which is not related to the CpG island pattern. 

### 2.2. Aconitase Activity and Expression

**Figure 2:**
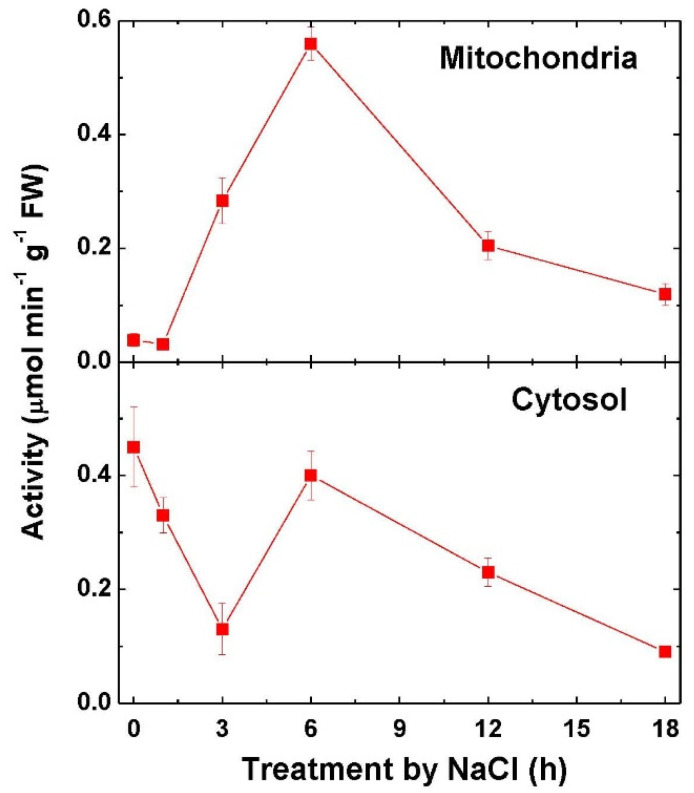
Change in activity of aconitase in the mitochondrial and cytosolic fractions after the transfer of maize seedlings to 150-mM NaCl. The data represent the means of three biological repeats ± SD. The control (untreated) samples of activities did not exhibit statistically significant changes in the variation level during the experiment.

The profile of the dynamics of aconitase activity in the leaves of maize plants exposed to salt stress has its specific features for the mitochondrial and cytosolic forms. The mitochondrial aconitase activity increased until 6 h of exposition to salt stress, following its further decline to 18 h; however, the activity remained higher than before the salt treatment. The activity of the cytosolic aconitase exhibited a decrease upon the exposition to salt stress, with some elevation (but below the control level) at 6 h of incubation, followed by its further decline (Figure 2). 

**Figure 3:**
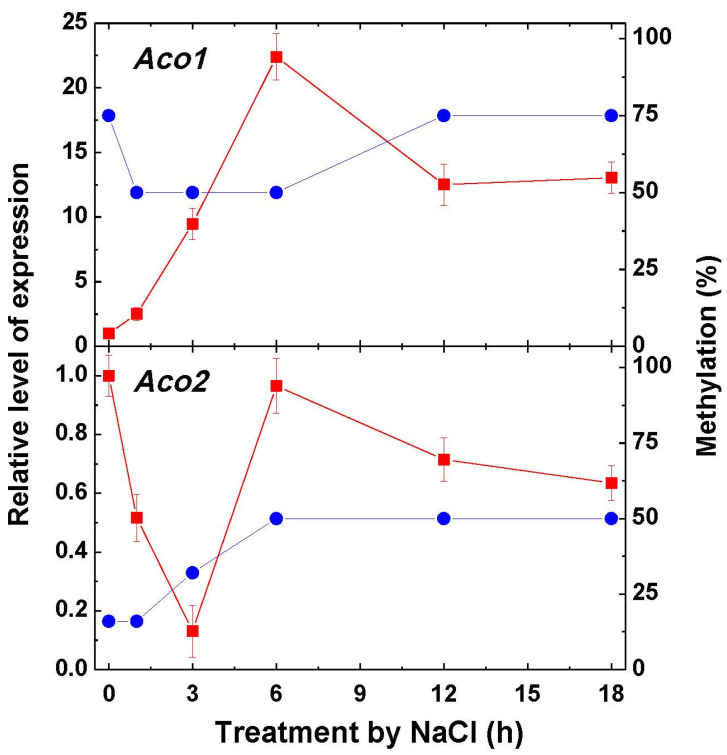
Relative level of transcripts of the genes *Aco1* and *Aco2* and the rate of methylation of their promoters in maize leaves in the course of incubation of maize seedlings in 150-mM NaCl. The data represent the means of three biological repeats ± SD. The control (untreated) samples of gene expression values did not exhibit statistically significant changes in the variation level.

The expression of the aconitase genes *Aco1* and *Aco2* encoding, correspondingly, the mitochondrial and cytosolic forms [27] exhibits the marked increase of transcripts of *Aco1* at 6 h of NaCl incubation, followed by a decline (Figure 3). This corresponds to the decrease of methylation of the promoters of the gene *Aco1* immediately after the imposition to salt stress and to the return to the original level of methylation at 12 h of incubation. The methylation pattern of the gene *Aco2* encoding the cytosolic form gradually increased during 6 h of incubation and then stabilized. The expression of *Aco2* decreased immediately upon incubation and then increased at 6 h, followed by a further decrease (Figure 3). 

### 2.3. Fumarase Activity and Expression 

**Figure 4:**
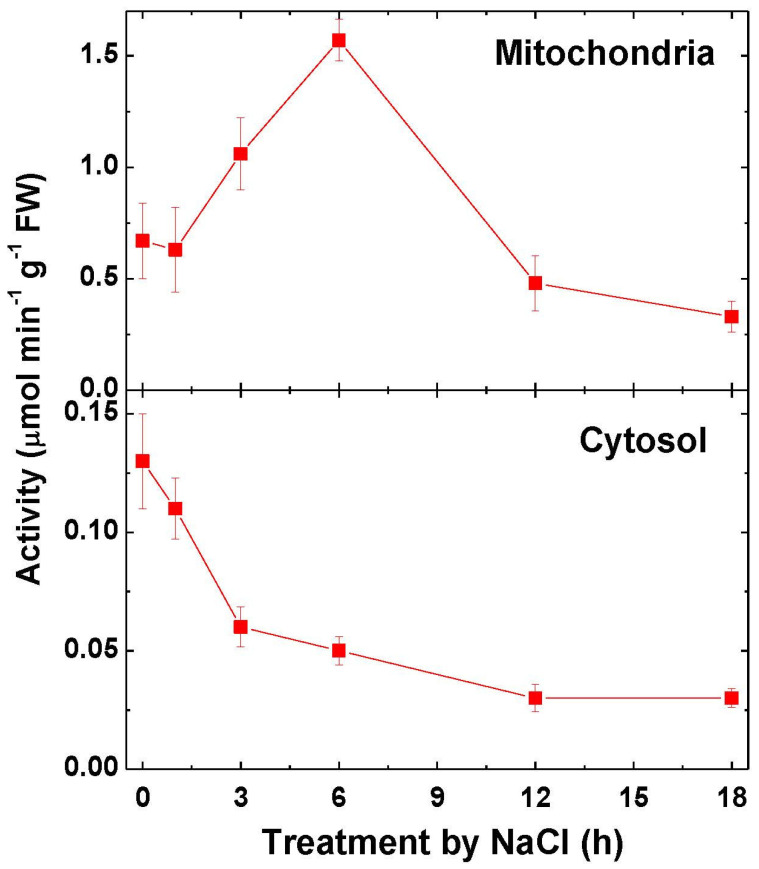
Change in the activity of fumarase in the mitochondrial and cytosolic fractions after the transfer of maize seedlings in 150-mM NaCl. The data represent the means of three biological repeats ± SD. The control (untreated) samples of the values of activity did not exhibit statistically significant changes in the variation level.

The fumarase activity in mitochondria increased during 6 h of incubation with NaCl by 2.5 times and then declined (Figure 4). In the cytosol, the fumarase activity exhibited a continuous decrease upon salt stress, down to four-fold lower values at 12 h of incubation as compared to the initial level.

**Figure 5:**
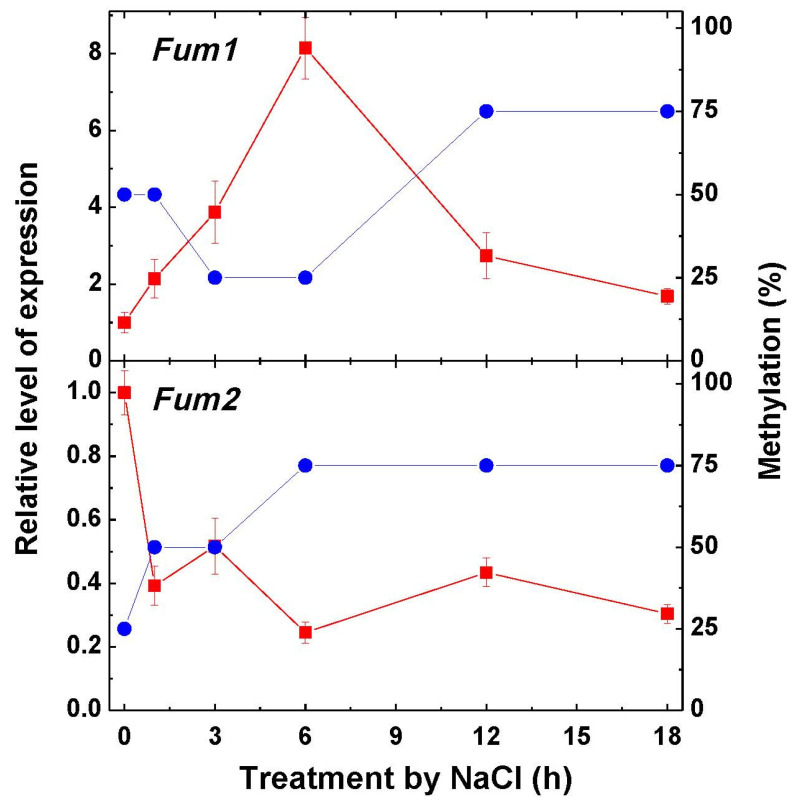
Relative level of transcripts of the genes *Fum1* and *Fum2* and the rate of methylation of their promoters in maize leaves in the course of incubation of maize seedlings in 150-mM NaCl. The data represent the means of three biological repeats ± SD. The control (untreated) samples of gene expression values did not exhibit statistically significant changes in the variation level.

The expression of fumarase genes *Fum1* and *Fum2* encoding, correspondingly, the mitochondrial and cytosolic forms of the enzyme showed the increase of transcripts of *Fum1* during the first 6 h of NaCl incubation, followed by a decline (Figure 5). The decrease of methylation of the promoters of the gene *Fum1* was observed at 3–6 h of the imposition to salt stress; then, the degree of methylation increased. The methylation pattern of the gene of the cytosolic fumarase *Fum2* increased during 6 h of incubation and then stabilized. The expression of *Fum2* declined immediately upon incubation, remaining at a 3–5 times lower level during 3–18 h of incubation as compared to the initial level (Figure 5). 

### 2.4. Expression of ZmCOI6.1, Pif5 and Pif6

**Figure 6:**
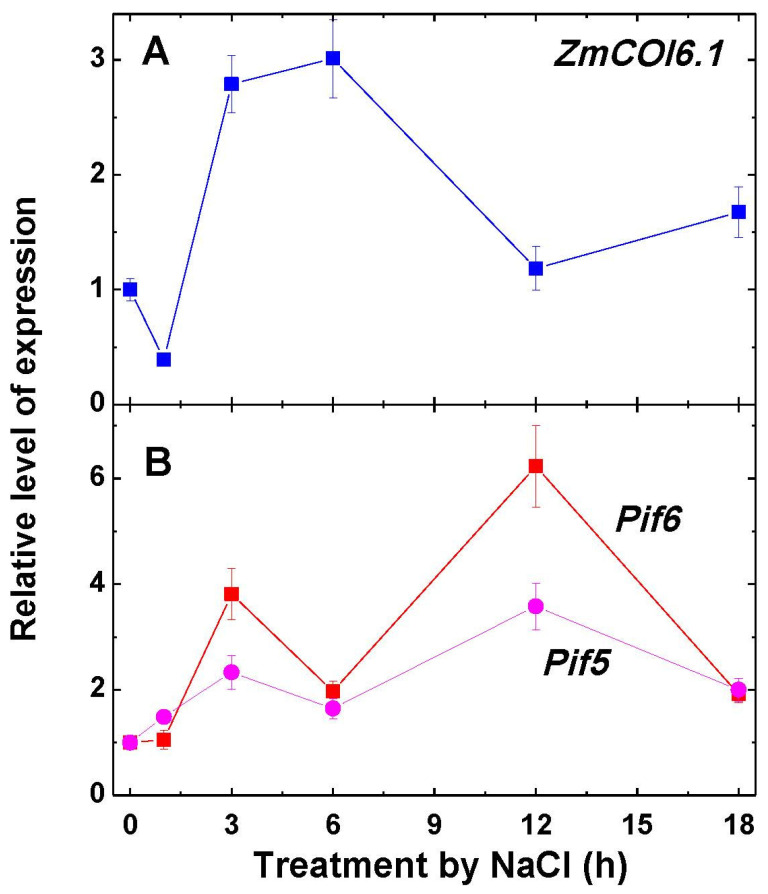
Relative level of transcripts of the gene *ZmCOI6.1* (**A**) and of the genes *Pif5* and *Pif6* (**B**) in maize leaves in the course of incubation of maize seedlings in 150-mM NaCl. The data represent the means of three biological repeats ± SD. The control (untreated) samples of gene expression values did not exhibit statistically significant changes in the variation level.

The NaCl-inducible gene *ZmCOI6.1*, used as a control of the salt stress response in the investigated plants, exhibited a three-fold increase in its expression at 3 to 6 h of salt stress; then, its expression declined to a level slightly higher than the initial degree of expression (Figure 6A). The expression of the genes *Pif5* and *Pif6* increased upon the imposition to NaCl, then decreased at 6 h, then increased again by 12 h, followed by a further decline (Figure 6B). 

## 3. Discussion

The phenomenon of “salt respiration”, which corresponds to the increase of respiration under early salt stress, has been known for many years [14]. Understanding the adaptive function of this respiratory increase comes from the major shifts in metabolism observed upon plant imposition to high concentrations of NaCl or other salts. Recently, it was shown that, under salt stress, the TCA cycle shifts to the operation of GABA shunt, bypassing the reactions of the 2-oxogutarate dehydrogenase complex and succinyl-CoA synthetase [19], and the pathways of electron transport non-coupled to ATP synthesis are induced [20,21]. These metabolic shifts result in the reorganizing of the metabolism that stimulates the active membrane transport processes and prevents harmful effects of elevated ion concentrations on plant functions. 

Many reactions of the TCA cycle are distributed between the mitochondria and cytosol. This refers to the equilibrium reactions catalyzed by aconitase and fumarase. While the changes in aconitase activity in both compartments are linked to the changes in the efflux of citrate from mitochondria [6,7,28], the alterations in the mitochondrial and cytosolic activities of fumarase may result in the exchange of malate and fumarate between the mitochondria, cytosol, and other compartments [1,2,3]. These changes can lead to the adjustment of plant metabolism during “salt respiration” and provide an efficient metabolic adjustment to salt stress conditions. 

In the current study, a differential expression of the mitochondrial and cytosolic forms of TCA cycle enzymes was investigated. The cytosolic aconitase may constitute up to 90% of its total activity in the plant cell [2]. The PCR analysis of the genes of the mitochondrial (*Aco1*) and cytosolic (*Aco2*) aconitase revealed a correlation between the activity of the mitochondrial from of aconitase and the expression of the gene *Aco1*. A similar correlation was observed for the cytosolic form of aconitase, with the tendency of a decline of the *Aco2* gene expression and the decrease of the cytosolic aconitase activity. Changes in the activity of differently localized fumarase isoforms were similar to those of aconitase isoforms. The distribution of fumarase activity between the mitochondria and cytosol in many organisms is approximately equal, or the mitochondrial form prevails, as in the current study [29]. The study of the dynamics of gene expression of the mitochondrial and cytoplasmic forms of fumarase showed that the detected changes in the enzyme activity are determined at the genetic level by the expression of corresponding genes. The expression of aconitase and fumarase genes is regulated by the methylation of promoters of the genes encoding the mitochondrial and the cytosolic forms of fumarase. A similar mechanism of regulation of TCA cycle enzymes was shown earlier for the anoxic conditions [30,31,32]. 

Although the relative expression of *Aco1* was much higher than that of *Aco2* in the course of incubation in 150-mM NaCl (Figure 3), the enzyme activity of Aco1 in the mitochondria was not much higher than that of Aco2 in cytosol (Figure 2). The increase of *Aco1* expression under salt stress and corresponding lower change of activity as compared to the control values may be related to the different levels of synthesis and degradation of the aconitase protein in mitochondria as compared to the cytosol. Previously, it was shown that the mitochondrial aconitase is more susceptible to degradation by reactive oxygen and nitrogen species via their effects on the Fe-S clusters of the enzyme, which can result in the decrease of its activity [33]. The observed certain discrepancy between the expression and the level of methylation reflects the fact that the expression is determined not only by methylation but, also, by other factors [34]. We can hypothesize that the observed non-returning of methylation to the original level is connected with the physiological state of plants and assume that, at a later stage, the methylation level would return to the original value.

The observed increase in expression of the genes encoding the mitochondrial forms of aconitase and fumarase corresponds, in general, to the pattern of increase of the level of transcripts of *ZmCOI6.1* (Figure 6A) that serves as the gene of a general response to stress [35]. This indicates that the activation of this marker gene and of the genes encoding the mitochondrial forms of aconitase and fumarase may be under the same control mechanism, which underlies the phenomenon of “salt respiration”. It is known that phytochrome interacting factors (PIFs) play an important role in establishing a stress response at the metabolic level via the regulation of the expression of the genes participating in the biosynthesis and metabolism of abscisic acid (ABA), which regulates the adaptation of plants to cold, drought, salinity, and other stress factors. PIFs can function in signal transduction cascades by integrating multiple signals for the transcription of stress-related genes [36]. The pattern of expression of two genes encoding PIFs (*Pif5* and *Pif6*) indeed show a response to NaCl imposition (Figure 6B); however, the pattern of their expression is different from the patterns of *ZmCOI6.1*, *Aco1*, and *Fum1*. This may indicate that they are not directly involved in the regulation of aconitase and fumarase during the stress response, and their function may be connected mostly to the regulation of TCA cycle enzymes by light, as shown earlier [27,37,38]. 

## 4. Materials and Methods

### 4.1. Object of Investigation

The two-week-old seedlings of maize (*Zea mays* L., cv Voronezhskaya 76, obtained from the Voronezh branch of the All-Russian Research Institute of Maize) were used in this study. The plants were grown hydroponically without the addition of mineral nutrients at 25 °C and at 12 h of daylight of 25 W m^−2^ supplied by luminescent lamps. The salt stress was modeled by the incubation of seedlings in a 150-mM NaCl solution. This concentration was shown to be sufficient for inducing changes in the metabolism without severe damage of the plants [39]. The seedlings exposed in water served as a control. The plant samples were taken for experiments at 0, 1, 3, 6, 12, and 18 h of exposition to salt stress.

### 4.2. Determination of Aconitase and Fumarase Activities 

Aconitase and fumarase activities were monitored spectrophotometrically at 240 nm. The unit of activity corresponded to the amount of the enzyme forming 1-µmol *cis*-aconitate or fumarate per minute. For aconitase measurements, citrate was used as a substrate. The increase in the optical density was detected at 240 nm at 25 °C due to the formation of the double bond in *cis*-aconitate. The assay medium contained 100-mM tris–HCl buffer, pH 8.0, 50-mM trisodium citrate, and 2-mM EDTA. The extinction coefficient of *cis*-aconitate 3.6 mM^−1^ cm^−1^ was used for the calculations of the activity [4]. The medium for the fumarase assay contained 50-mM tris-HCl buffer, pH 7.6, 20-mM D,L-malate (sodium salt), 2-mM EDTA, and 5-mM MgCl_2_. The extinction coefficient of fumarate 2.44 mM^−1^ cm^−1^ [40] was used for the calculations. The total protein was assayed by the method of Lowry et al. [41]. 

### 4.3. Subcellular Distribution of Aconitase and Fumarase

Separation of the cytosolic and mitochondrial activities of aconitase and fumarase was performed at 0–4 °C. Five grams of leaf tissue was homogenized in 25 ml of 20-mM HEPES-KOH buffer, pH 7.6, containing 0.25-M sucrose, 40-mM mannitol, 5-mM MgCl_2_, 1-mM EDTA, and 5-mM DTT and filtered through four layers of cheesecloth. After the first centrifugation at 1300× *g* for 5 min, the debris of the cell walls was discarded, and the supernatant was centrifuged again at 15,000× *g* for 20 min. The supernatant was considered a cytosolic fraction. The mitochondrial pellet (contaminated with peroxisomes and other organelles) was ruptured in the same buffer without sucrose but containing 0.01% Tween 80, homogenized, and recentrifuged in the same conditions, and the supernatant was used for determination of the mitochondrial aconitase and fumarase activities. Further separation of the organelles by isopycnic centrifugation was not performed, because aconitase is localized in the mitochondria and cytosol and absent from other organelles (e.g., peroxisomes) [4]. The cross-contamination between the mitochondrial and cytosolic fractions did not exceed 6%, which was determined by measuring the activities of lactate dehydrogenase and succinate dehydrogenase, as described earlier [3,4]. 

### 4.4. RNA Isolation and PCR Analysis 

The total cellular RNA was isolated using guanidine–thiocyanate–chloroform extraction using LiCl for sedimentation. RNA concentration in the sample was determined on the T70+ UV-VIS spectrophotometer (PG Instruments Limited, Leicestershire, UK). Reverse transcription of RNA was performed using the reverse transcriptase MMULV (SibEnzyme, Novosibirsk, Russia), according to the manufacturer’s recommendations. Real-time PCR was performed using LightCycler 96 (Roche Applied Science, Indianapolis, IN, USA) using SYBR Green for staining. The primers were designed using the software Primer3 [42] (Table 1). Amplification parameters: preliminary denaturation 95 °C for 5 min, then the cycle: 95 °C for 30 s, 58–63 °C for 30 s, and 72 °C for 30 s (detection). The amount of matrix was controlled by the parallel amplification of the elongation factor Ef-1α and 18S with gene-specific primers [43]. The total RNA without the stage of reverse transcription was used as a negative control. The relative level of expression of the analyzed genes was determined by the 2^−^^ΔΔCT^-method [44] using LightCycler 96 Software Version 1.1 (Roche Applied Science, Indianapolis, IN, USA).

For the analysis of the promoters of the genes for the presence of CpG islands and for the design of methyl-specific primers, the program MethPrimer (LiLab, UCSF, San Francisco, CA, USA, http://www.urogene.org/methprimer/, accessed on 8 February 2018) [45] was used. The nucleotide sequence of the promoter region of fumarase was taken from the NCBI database. The polymerase chain reaction with methyl-specific primers was performed using an AmpliSence kit (Helicon, Moscow, Russia). The PCR reaction was performed on a Tertsik instrument (DNA Technology, Protvino, Russia) using the following amplification parameters: initial denaturation at 95 °C for 5 min, then 35 cycles: 95 °C for 20 s, 55–60 °C for 20 s, and 72 °C for 30 s. For the selection of primers for methyl-specific PCR, the MethPrimer program [45] was used; see Table 2 for the full set of methyl-specific primers.

The analytical electrophoresis of PCR products was conducted in 1% agarose gel (Helicon, Russia). The results were recorded at 312 nm using a transilluminator and DNA Analyzer (DNA-Technology, Moscow, Russia) and analyzed using the program Gel Explorer, version 1.0 (DNA-Technology, Moscow, Russia). The calculation of quantitative values of methyl-specific PCR was implemented on the basis of electrophoregrams of PCR products. The quantitative parameters of the degree of promoter methylation represent the total result of PCR analysis of the studied CG dinucleotides in the promoter of the investigated gene. 

### 4.5. Statistical Analysis

All experiments were repeated three to five times, and analytic assays in each sample were taken three times. The data in the figures were the means of three biological repeats ± SD. The statistically significant differences at *p* < 0.05 were discussed.

## 5. Conclusions

This study unequivocally showed that salt stress differentially affects the expression of the genes encoding the mitochondrial and the cytosolic forms of aconitase and fumarase. The obtained data also revealed that the mechanism of regulation of the synthesis of the fumarase and aconitase isoforms was connected with the methylation status of promoters of their genes. For both the mitochondrial and the cytosolic forms of aconitase and fumarase, the degree of promoter methylation and the expression of the corresponding genes and enzyme activities were intercorrelated. A high level of promoter methylation suppressed the expression of their genes. This means that the mitochondrial and cytosolic forms of aconitase and fumarase were regulated via the epigenetic mechanism in response to salt stress. The activation of the mitochondrial forms of aconitase and fumarase and the suppression of the cytosolic forms corresponded to the intensification of mitochondrial respiration in the first hour of exposition to salt stress, which explained the phenomenon of “salt respiration”. It corresponded to the previously reported activation of alternative oxidase and to the shift to a GABA shunt. At the same time, the cytosolic interconversion of TCA cycle intermediates was suppressed at the level of aconitase and fumarase, indicating a tighter connection of respiration to the mitochondria and a possible suppression of the malate and citrate valves in the condition of salt stress. This supports the idea that the mitochondria represent a central hub in the adaptation of plants to salt stress. 

## Figures and Tables

**Table 1:** Oligonucleotides for RT-PCR.

Gene	Primer	Sequence
*Aco1*	forward	5′-TGGAAGGAGATGCTGTCAGT-3′
	reverse	5′-CGTATAGCGCCATCCACATG-3′
*Aco2*	forward	5′-CAAGTTCTTCAGCCTTCCGG-3′
	reverse	5′-GCAAGGTCTACAACTGCTGG-3′
*Fum1*	forward	5′-GATTACTTCGATCATTGAGGT-3′
	reverse	5′-ACCAGAACTCGCGGATGTGGC-3′
*Fum2*	forward	5′-ACCAGAACTCGCGGATGTGGC-3′
	reverse	5′-TGGTTCATTCTCAGGCAGAGA-3′
*ZmCOI6.1*	forward	5′-GGGTGTTCCTCAAGTACGGG-3′
	reverse	5′-GGGTGGGTACGGTAGCAAAA-3′
*Pif5*	forward	5′-GAAAGAACCTTTCCGCGTGT-3′
	reverse	5′-AAAGCAGGGCATGTGAACAG-3′
*Pif6*	forward	5′-TGAATCCAGGTTGCATCCCT-3′
	reverse	5′-CATCCCTGAGGTGACCGATT-3′

**Table 2:** Oligonucleotides for methyl-specific PCR.

Gene	Position of the Studied Cytosine		Primer	Sequence
*Aco1*	I	-425 bp	forward M	5′-TGTATTTTAGAAATAGAGTTTTGCGT-3′
			reverse M	5′-CTACGCACGAATTAAATTCGAAT-3′
			forward U	5′-ATGTATTTTAGAAATAGAGTTTTGTGT-3′
			reverse U	5′-ATACCTACACACAAATTAAATTCAAA-3′
	II	-565 bp	forward M	5′-TAGAGATTATTTTTCGATTCGATTC-3′
			reverse M	5′-CTACGCACGAATTAAATTCGAAT-3′
			forward U	5′-GAGATTATTTTTTGATTTGATTTGGTT-3′
			reverse U	5′-ATACCTACACACAAATTAAATTCAAA-3′
	IIII	-941 bp	forward M	5′-TTATTAACGTTTAGCGGTAGAGTTC-3′
			reverse M	5′-CTACGCACGAATTAAATTCGAAT-3′
			forward U	5′-ATTAATGTTTAGTGGTAGAGTTTGT-3′
			reverse U	5′-ATACCTACACACAAATTAAATTCAAA-3′
*Aco2*	I	-579 bp	forward M	5′-ATTCGAAAATAGTGAGAAGTTGTC-3′
			reverse M	5′-TAAAAAACTAACTAACCAAATCGCT-3′
			forward U	5′-TTATTTGAAAATAGTGAGAAGTTGTTG-3′
			reverse U	5′-TCAAACAAACCAAAACATAATACATACA-3′
	II	-821 bp	forward M	5′-GTTTATTGTACGTATAAGGAAGC-3′
			reverse M	5′-TAAAAAACTAACTAACCAAATCGCT-3′
			forward U	5′-GTTTATTGTATGTATAAGGAAGTGG-3′
			reverse U	5′-TCAAACAAACCAAAACATAATACATACA-3′
	III	-1034 bp	forward M	5′-GGTATTATAAAATTTATTAAGGTCGG-3′
			reverse M	5′-TAAAAAACTAACTAACCAAATCGCT-3′
			forward U	5′-AGTTTGGTATTATAAAATTTATTAAGGTTG-3′
			reverse U	5′-TCAAACAAACCAAAACATAATACATACA-3′
*Fum1*	I	-778 bp	forward M	5′-TAGGAATAATTTAAATAATACGG-3′
			reverse M	5′-TCTATTATAAAATAATACTTTCCC-3′
			forward U	5′-GTAGGAATAATTTAAATAATATGG-3′
			reverse U	5′-TCTATTATAAAATAATACTTTCCC-3′
	II	-717 bp	forward M	5′-GGTAGGGAATGGTTTGCGT-3′
			reverse M	5′-TCTATTATAAAATAATACTTTCCC-3′
			forward U	5′-GGTAGGGAATGGTTTGTGT-3′
			reverse U	5′-TCTATTATAAAATAATACTTTCCC-3′
	IIII	-552 bp	forward M	5′-TTGAAGGTTATTTATTTATACGG-3′
			reverse M	5′-TCTATTATAAAATAATACTTTCCC-3′
			forward U	5′-ATTGAAGGTTATTTATTTATATGG-3′
			reverse U	5′-TCTATTATAAAATAATACTTTCCC-3′

## Data Availability

The datasets generated for this study are available upon request from the corresponding author.

## Abbreviations

GABA	gamma-aminobutyric acid
PIF	phytochrome interacting factor
RNS	reactive nitrogen species
ROS	reactive oxygen species
TCA cycle	tricarboxylic acid cycle
